# Novel Use of Proton Magnetic Resonance Spectroscopy (1HMRS) to Non-Invasively Assess Placental Metabolism

**DOI:** 10.1371/journal.pone.0042926

**Published:** 2012-08-10

**Authors:** Fiona C. Denison, Scott I. Semple, Sarah J. Stock, Jane Walker, Ian Marshall, Jane E. Norman

**Affiliations:** 1 MRC Centre for Reproductive Health, University of Edinburgh, Queen’s Medical Research Institute, Edinburgh, Lothian, United Kingdom; 2 Clinical Research Imaging Centre, University of Edinburgh, Queen’s Medical Research Institute, Edinburgh, Lothian, United Kingdom; 3 Simpson Centre for Reproductive Health, Edinburgh Royal Infirmary, Edinburgh, Lothian, United Kingdom; 4 School of Clinical Sciences, University of Edinburgh, Edinburgh, Lothian, United Kingdom; Otto-von-Guericke University Magdeburg, Germany

## Abstract

**Background:**

Placental insufficiency is a major cause of antepartum stillbirth and fetal growth restriction (FGR). In affected pregnancies, delivery is expedited when the risks of ongoing pregnancy outweigh those of prematurity. Current tests are unable to assess placental function and determine optimal timing for delivery. An accurate, non-invasive test that clearly defines the failing placenta would address a major unmet clinical need. Proton magnetic resonance spectroscopy (^1^H MRS) can be used to assess the metabolic profile of tissue *in-vivo*. In FGR pregnancies, a reduction in N-acetylaspartate (NAA)/choline ratio and detection of lactate methyl are emerging as biomarkers of impaired neuronal metabolism and fetal hypoxia, respectively. However, fetal brain hypoxia is a late and sometimes fatal event in placental compromise, limiting clinical utility of brain ^1^H MRS to prevent stillbirth. We hypothesised that abnormal placental ^1^H MRS may be an earlier biomarker of intrauterine hypoxia, affording the opportunity to optimise timing of delivery in at-risk fetuses.

**Methods and Findings:**

We recruited three women with severe placental insufficiency/FGR and three matched controls. Using a 3T MR system and a combination of phased-array coils, a 20×20×40 mm^1^H MRS voxel was selected along the ‘long-axis’ of the placenta with saturation bands placed around the voxel to prevent contaminant signals. A significant choline peak (choline/lipid ratio 1.35–1.79) was detected in all healthy placentae. In contrast, in pregnancies complicated by FGR, the choline/lipid ratio was ≤0.02 in all placentae, despite preservation of the lipid peak (p<0.001).

**Conclusions:**

This novel proof-of-concept study suggests that in severe placental insufficiency/FGR, the observed 60-fold reduction in the choline/lipid ratio by ^1^H MRS may represent an early biomarker of critical placental insufficiency. Further studies will determine performance of this test and the potential role of 1H-MRS in the *in-vivo* assessment of placental function to inform timing of delivery.

## Introduction

Placental insufficiency is one of the commonest causes of fetal growth restriction (FGR) and antepartum stillbirth. When placental insufficiency is diagnosed antenatally, the only effective treatment is delivery which, if preterm is itself associated with increased morbidity and mortality and considerable financial costs. [1] If placental insufficiency remains undiagnosed and results in stillbirth [2], this can have profound and long lasting consequences for parents and their extended family. [3] One of the main challenges in current obstetric practice is therefore our inability to accurately and non invasively diagnose placental insufficiency, quantify its severity and predict its clinical sequelae. Better diagnosis would improve the timing of clinical interventions and potentially improve perinatal outcome.

In current clinical practice, diagnosis of placental insufficiency and fetal compromise is largely based on Doppler assessment of umbilical artery blood flow, fetal arterial and venous Dopplers (e.g. ductus venosus and middle cerebral artery Doppler waveforms) or ultrasound biometry. [4] Although abnormal Dopplers correlate with cord pH, fetal hypoxia and lactate generation [5], and are associated with the presence of gross placental lesions detectable by ultrasound [6], [7], neither Doppler nor conventional ultrasound is able to directly measure placental function. Sibley *et al* [8] recently proposed that the constellation of physiological and morphological changes may constitute a placental phenotype in some pregnancies complicated with FGR with an abnormal phenotype being associated with poor perinatal outcome. Development of non-invasive tools capable of assessing placental metabolism and cell turnover directly may allow placental phenotyping to occur, thus enabling clinical interventions to be targeted to those pregnancies at highest risk of adverse outcome and timely delivery to be effected.

Magnetic resonance imaging (MRI) is a non-invasive imaging technique which is safe in pregnancy. The technique has the ability to acquire a combination of anatomical information with high spatial resolution, and directly assess a variety of physiological function. MRI is non-invasive and does not involve the use of ionizing radiation, making it an ideal candidate for the development of biomarkers for fetal compromise.

Until recently the use of MRI for placental assessment has been primarily used as a complementary technique to ultrasound, for example assessing placental invasion in cases of suspected placenta accreta. [9] However, with technological advances it has been proposed that advanced placental MRI imaging could provide biomarkers for disease onset and outcome. Recent studies demonstrate that FGR is associated with a reduction in MRI measures of proton diffusion within the placenta, and changes in placental volume and thickness [10], thus supporting this concept.

Proton magnetic resonance spectroscopy (^1^H MRS) is based on similar principles to MRI. It uses a static magnetic field to temporarily align the nuclear magnetization of protons within the body. Radiofrequency pulses are then applied which give the protons enough energy to alter this alignment and, as the protons return to their original state, the resulting radiofrequency signal is detected by the MR system. [11] Whilst MRI produces information as an image, ^1^H MRS instead provides data on the relative concentrations of specific metabolites contained within selected regions of interest, with the local chemical and magnetic environment in which each proton is situated within that region determining the ‘chemical shift’ in resonant frequency that each proton’s signal exhibits after excitation. This chemical shift data is expressed as a frequency spectrum with contributions of specific metabolites appearing as individual peaks at discrete frequencies. The area under each frequency peak is proportional to the number of protons, and hence the concentration, of the metabolite. ^1^H MRS therefore has the potential to be a powerful, non-invasive method of assessing the metabolic profile of tissue *in vivo*.

In healthy pregnancies, a choline peak detected by ^1^H MRS is associated with a normal high degree of cell-turnover in the developing fetal brain whilst an increasing N-acetylaspartate (NAA) peak with gestational age reflects the increase in the number of neurons during brain development. In FGR pregnancies, reduction in NAA/choline ratio is emerging as a biomarker of impaired neuronal metabolism. [12] Although *in vitro* studies suggest that altered placental metabolism and reduced cell turnover may precede the onset of placental and intrauterine hypoxia [13], [14], we are not aware of any studies which have attempted to assess the metabolic footprint of the placenta in normal pregnancy and those complicated by FGR using ^1^H MRS.

The aim of our study was therefore to establish the feasibility of undertaking ^1^H MRS of the placenta *in vivo*, and to undertake the first proof-of-concept study to assess whether the metabolic footprint of the placenta differs in placenta from pregnancies complicated by FGR compared to healthy controls.

## Methods

### Ethics Statement

The study was approved by Lothian Research Ethics Committee (10/S1103/36) and all participants gave written, informed consent.

### Study Population

Patients were enrolled at the Simpson Centre for Reproductive Health at the Royal Infirmary, Edinburgh, UK. Magnetic resonance imaging (MRI) studies were performed at the Clinical Research Imaging Centre in the Queen’s Medical Research Institute, University of Edinburgh, Edinburgh, UK.

We studied 3 women with a singleton pregnancy complicated by severe FGR and suspected fetal compromise and 3 gestation matched controls. Gestation was calculated from the last menstrual period and confirmed by routine ultrasonography at 11–13 weeks gestation. All participants had a structurally normal fetal anomaly scan at 20 weeks gestation. Severe FGR was defined as an abdominal circumference by ultrasound <5^th^ centile. [15] Suspected fetal compromise was defined as absent or reversed end-diastolic flow on umbilical artery Doppler. Exclusion criteria for study participation included significant co-existing maternal systemic disease including gestational diabetes, microvascular disease, multiple pregnancy, or contraindication to MRI.

### Magnetic Resonance Studies

All magnetic resonance (MR) studies were performed using a wide-bore dedicated clinical research 3 tesla MR Verio system (Siemens Medical, Germany). Women were scanned in a left-lateral tilt to avoid compression of vena-cava with blood pressure constantly monitored using a Veris MR Vital Signs Monitor (Medrad, UK). Total MR acquisition times were limited to 40–45 minutes per participant. No fetal sedation was used. A combination of body and spine matrix phased-array coils was used to obtain all images and ^1^H MRS data. Prior to acquisition of ^1^H MRS data, a series of 2D HASTE slices was acquired in three orthogonal planes centred on the placenta. Multiple 6 mm slices were acquired with no inter-slice gap in all three planes, in order to localise the extent of the placenta. Each HASTE image took approximately 1 second to acquire, so all images were acquired with the mother free-breathing.

### MR Spectroscopy Studies

**Figure 1:**
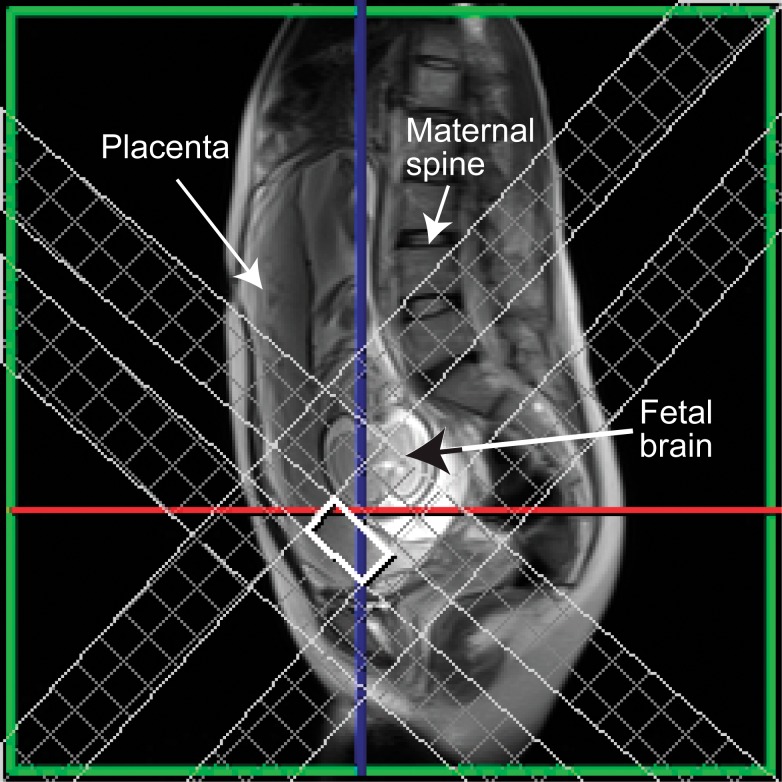
Voxel placement in the placenta with demonstration of saturation bands.

For ^1^H MRS acquisition, a 20×20×40 mm PRESS voxel was selected along the ‘long-axis’ of the placenta (TE/TR 144/1500 ms, 96 averages). The voxel was selected approximately 2 cm from the cord insertion in all cases and voxel selection was confirmed to be limited to within the placenta using the range of orthogonal HASTE slices (Figure 1). Six saturation bands were placed around the voxel to further prevent any non-placenta contaminant signals and a second-order semi-automatic shim was applied over the selected voxel to counter any local inhomogeneities in the magnetic field. The voxel dimensions were selected to maximise sampling of the placental unit, thereby increasing signal-to-noise values of the resulting spectra. ^1^H MRS data was acquired with the mother free-breathing. In our experience, the placental unit does not significantly move outwith our selected voxel dimensions during either maternal breathing, or fetal motion. The resulting raw spectral data was exported to an external workstation and MRS analysis to assess placental metabolism (Lipid/choline ratio) was quantified using the Java-based MRS analysis tool JMRUI (http://www.mrui.uab.es/mrui).

### Data Analysis

The ^1^H MRS quantification process was performed using the nonlinear least-squares quantitation algorithm AMARES (Advanced Method for Accurate, Robust and Efficient Spectral fitting) with peak fitting performed assuming a Lorentzian line shape. Since only two peaks were clearly identified, each peak was identified manually according to its frequency and the line widths and areas under the curves semi-automatically estimated. Birth percentiles were calculated using centile charts for birthweight for gestational age for Scottish singleton births. [16] Data were analysed by GraphPad Prism (Version 5.0).

## Results

### Demographics of Study Population

Maternal age ranged between 20 and 37 (mean, 28±6.8 years) and maternal body mass index (BMI) between 18.4 and 32.8 (mean, 25.7±4.8 kg/m^2^). The demographics of the study population at the time of MRS scan and delivery are demonstrated in Tables 1 and 2. Neonatal outcome and time between ^1^H MRS and delivery are demonstrated in Table 3.

**Table 1:** Maternal demographics, gestational age and antenatal Dopplers at the time of ^1^H MRS.

Subject	Age	Parity	BMI	Abdominal circumference ultrasound centile	Umbilicalartery	Liquorvolume	Gestational age at ^1^H MRS (wks^+days^)
Healthy 1	33	0+0	18.4	25^th^–50^th^	Normal	Normal	24^+4^
Healthy 2	31	1+0	23.1	50^th^–95^th^	Normal	Normal	30^+3^
Healthy 3	37	2+1	25.3	50^th^–95^th^	Normal	Normal	28^+0^
Compromised 1	30	0+0	23.1	<5^th^	AEDF	Reduced	28^+5^
Compromised 2	20	0+1	26.4	<5^th^	AEDF	Reduced	25^+0^
Compromised 3	23	0+1	32.8	<5^th^	AEDF	Reduced	27^+1^

AEDF (absent end diastolic flow).

**Table 2:** Characteristics of population at delivery.

Subject	Gestational age at delivery, wks^+days^	Mode delivery	Sex	Fetal weight (g)	Percentile at birth
Healthy 1	40^+0^	SVD	Male	3060	25^th^
Healthy 2	40^+3^	SVD	Male	3760	50^th^
Healthy 3	39^+5^	SVD	Male	3630	50^th^
Compromised 1	30^+4^	SVD	Male	750	<0.4^th^
Compromised 2	25^+4^	EmCS	Male	670	9^th^
Compromised 3	28^+6^	EmCS	Male	530	<0.4^th^

SVD (spontaneous vertex delivery), EmCS (emergency caesarean section).

**Table 3:** Neonatal outcome and choline/lipid integral 1H MRS ratio.

Subject	Outcome	Time between 1H MRS and delivery (days)	Choline/lipid integral ratio
Healthy 1	Alive and well	108	1.35
Healthy 2	Alive and well	70	1.79
Healthy 3	Alive and well	82	1.36
Compromised 1	Stillbirth^1^	13	<0.02
Compromised 2	Neonatal death at 42 days	4	<0.02
Compromised 3	Discharged from NNU with BPD and ROP on supplemental oxygen at 42^+4^ weeks corrected gestation	12	0.02

1 Compromised 1 pregnancy was expectantly managed until antenatal stillbirth occurred.

NNU (neonatal unit), BPD (bronchopulmonary dysplasia), ROP (retinopathy of prematurity).

### MRI Results

**Figure 2:**
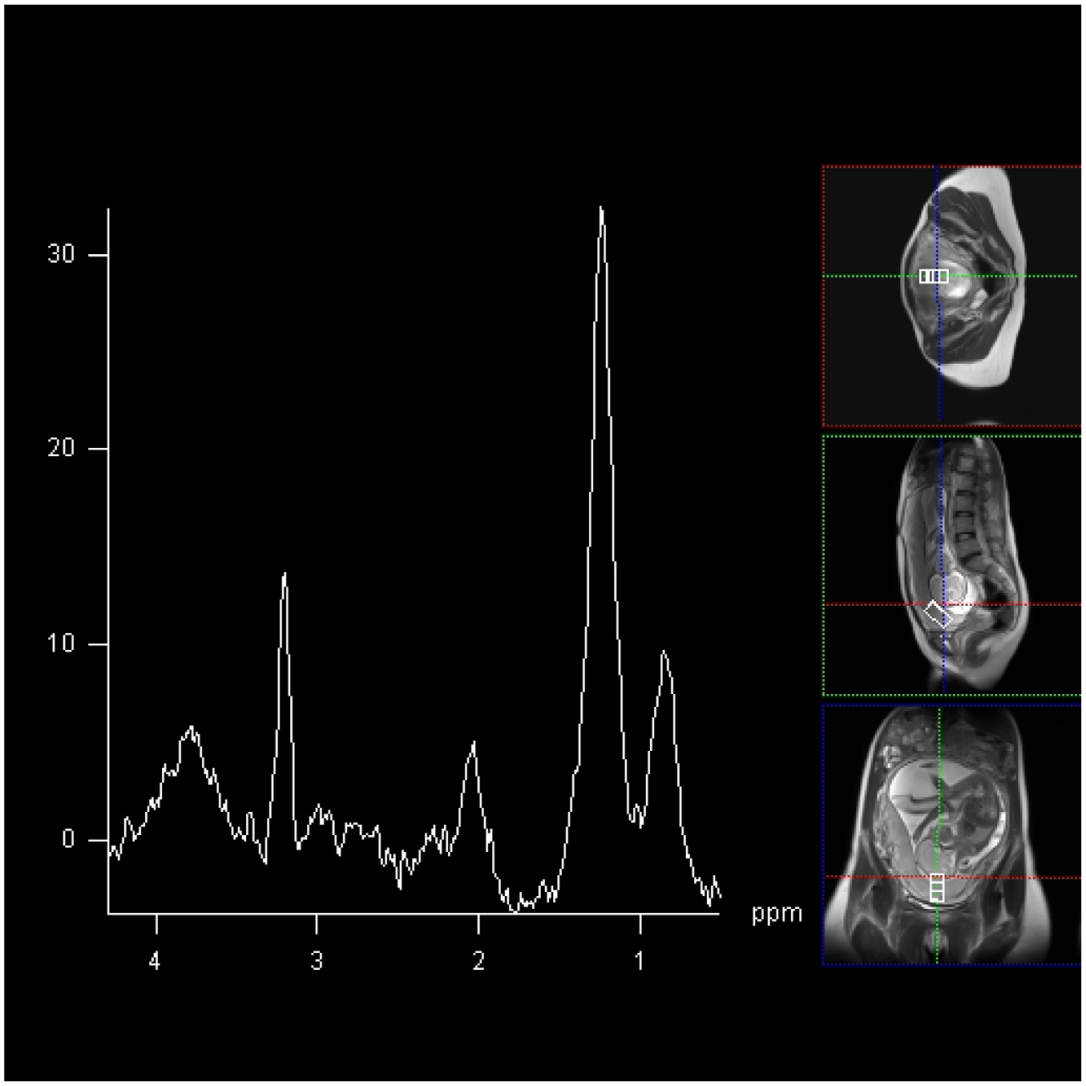
2×2×4 cm voxel MRS acquired at 144 ms from placenta from healthy participant 1. In this case, choline and lipid spectral peak demonstrated at frequencies of 3.21 ppm, and 1.3 ppm and 0.9 ppm per million (ppm) respectively.

**Figure 3:**
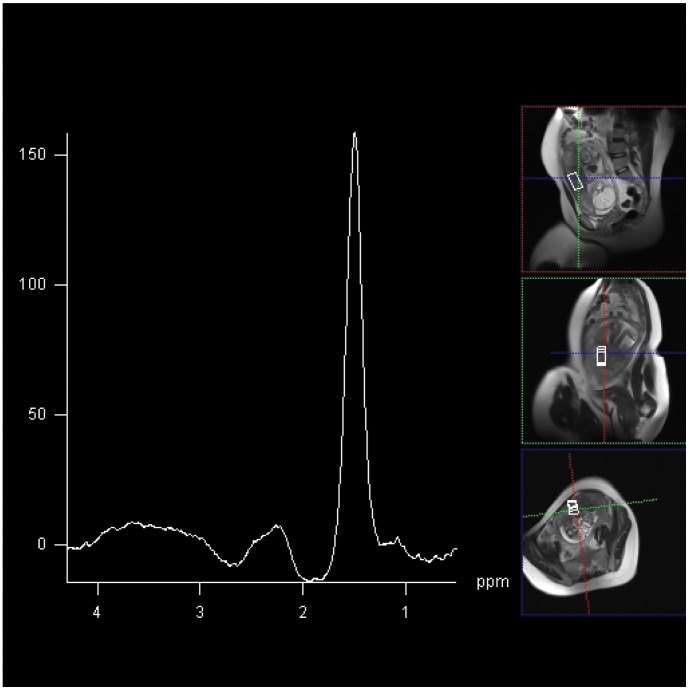
2×2×4 cm voxel MRS acquired at 144 ms from placenta from compromised participant 2. Lipid spectral peak demonstrated at frequency of 1.42 ppm. Choline peak below level of reliable detection.

*In utero* ^1^H MRS of the placenta was obtained in all participants. A choline and lipid peak were easily detectable, centred at 3.2 ppm and 1.2 ppm, respectively from placentae in all healthy controls (Figure 2). In the healthy controls a significant choline signal was obtained, resulting in a choline/lipid ratio of 1.35–1.79. In contrast, despite preservation of the lipid peak, there was severe attenuation or absence of detectable choline peak in placentae from pregnancies complicated by severe FGR and suspected fetal compromise (Figure 3). The choline/lipid ratio was reduced to ≤0.02, a reduction of more than 60-fold in pregnancies complicated by severe FGR compared to the gestation matched healthy controls (Table 3) (p<0.001).

## Discussion

To our knowledge, this is the first report of placental ^1^H MRS *in vivo* in normal and FGR pregnancies. We demonstrate that in healthy pregnancies, choline and lipid spectral peaks were clearly detected in all placenta using ^1^H MRS. In contrast, in pregnancies complicated by FGR, despite preservation of the lipid peak, the choline peak was severely attenuated or absent from all placentae. We speculate that a reduction in the choline/lipid ratio by ^1^H MRS may provide a novel biomarker of critical placental failure, indicative of reduction in cell turnover, which predates fetal hypoxia and antepartum stillbirth in pregnancies with severe FGR.

To date, knowledge about placental function in FGR pregnancies has been largely extrapolated from *in vitro* and *ex vivo* studies. Placental weight, total volume, villous volumes and surface area are significantly reduced in FGR pregnancies. [17] At a histological level, there is evidence of increased apoptosis, a thickened basal lamina and reduction in cytotrophoblastic nuclei and cell-turnover. [14], [18] The marked impairment of nutrient transport [13], [19] and placental perfusion which occurs results in global placental dysfunction and altered metabolism [20], [21]. Cetin *et al* suggested that such alterations in placental metabolism and function may precede the onset of placental and intrauterine hypoxia in affected pregnancies. [13] If it were possible to detect altered placental metabolism prior to the onset of critical placental failure, this might afford an opportunity for more timely clinical intervention (including delivery) thus preventing adverse perinatal outcome.

A variety of non-invasive methods have attempted to assess placental function *in vivo* to predict the functional capacity of the pregnancy and/or pregnancy outcome. The ultrasound based ‘Grannum grading’ of placenta, which was originally developed as a biomarker for fetal lung maturity, has been assessed as a predictive tool for fetal growth restriction [22] and placental function [23]. However, although there is a relationship between Grannum III grade and FGR, the positive predictive and sensitivity value of this “test” is low (62% and 66%, respectively) [22] and Grannum grading at 31–34 weeks of gestation is unable to reliably predict the functional capacity of the term placenta as expressed by the surrogate measure, morphometric diffusive conductance. [23] More recently, near infrared spectroscopy has been explored as potential method of assessing tissue oxygenation and placental function. [24]–[26] To date, results have been conflicting in FGR pregnancies with tissue oxygenation indexes exhibiting both increase and decrease in the presence of FGR depending on its cause. [24]–[25] Furthermore, due to technical limitations, the latter technique is only able to assess placental oxygenation within a narrow range and in women with an anterior placentae and a thin layer of subcutaneous fat. [24] Until further method development occurs, these techniques are therefore unlikely to have a clinical utility in quantifying placental oxygenation and function *in vivo*.

Several groups have used MRI as a tool for assessing fetal and placental structure and function *in vivo.* At the macroscopic level, placental volume measured by MRI during the second trimester correlates with uterine artery perfusion and is reduced in pregnancies that subsequently delivered FGR infants. [27] Furthermore, the severity of FGR and incidence of fetal or neonatal mortality has been shown to correlate with the MR volume of placenta affected by pathology. [28] More recently Wright et al demonstrated that placental relaxation times (T1 and T2) were negatively correlated with gestation. [29] However, when the relaxation times were compared to postnatal examination, T2 only correlated with placental fibrin deposition if the scan and delivery were within one week of each other.

Studies using MRI to assess placental function are more limited. Bonel *et al* report that reduced apparent diffusion coefficient (ADC) as measured by diffusion-weighted MRI is exhibited in placental dysfunction associated with FGR. The authors hypothesised that placenta dysmaturity and focal disruption of the placental barrier which occur in FGR was responsible for the altered diffusion [30]. Using the technique of intravoxel incoherent motion and perfusion fraction mapping, Moore *et al* identified differences in function within the normal placenta in vivo, and between the placentae of normal and IUGR pregnancies. [31] Finally, using gadoterate melamine for contrast enhancement, Brunelli *et al* demonstrated that intervillous circulation was severely compromised in pregnancies with severe FGR. [32] However, this study was undertaken only a few hours prior to delivery by caesarean section due to concerns about fetal toxicity of gadolinium-based contrast agents, which are not licenced for use in pregnancy. None of these MRI techniques assess placental metabolism and function directly.

^1^H MRS by comparison to MRI is able to dynamically assess levels of specific metabolites in the region of interest selected. To date, the use of ^1^H MRS during pregnancy has been restricted to assessment of the fetal brain in health and disease. In pregnancies complicated by FGR, a reduction in NAA/choline in the fetal brain is thought to be indicative of impaired neuronal metabolism and reduced cell-turn over [12], and the presence of lactate methyl [33] to be indicative of established fetal hypoxia. However, both of these ‘biomarkers’ are likely to develop relatively late in the evolution of fetal compromise and hypoxia, limiting opportunities for therapeutic intervention.

To our knowledge, ^1^H MRS has not been previously undertaken in the placenta *in vivo*. We demonstrate that in FGR pregnancies with suspected compromise, despite preservation of the lipid peak, there is a severe reduction or absence of a placental choline peak. This is in contrast to healthy pregnancy where choline and lipid peaks are readily detectable. The presence of choline by ^1^H MRS in organs including the fetal brain is thought to indicate cell-turnover and growth. [34] In contrast, a reduction in the choline peak (compared to baseline) occurs when cell turnover is significantly reduced and in the presence of apoptosis. [35] Using *ex vivo* and *in vitro* models, Heazell *et al* [36] and others [18] [14] have demonstrated a reduction in cell-turnover and increase in apoptosis in placentae from pregnancies with FGR. We therefore propose that the significant reduction in choline/lipid ratio which we demonstrate in FGR placentae may be a novel biomarker of reduced cell turnover before apoptosis resulting in impaired placental function and critical organ failure.

Acquiring the ^1^H MRS data at 3T had the added benefit of the increased signal to noise available at this higher clinical field strength. This meant that less averages were required to obtain an acceptable signal-to-noise ratio for our ^1^H MRS data. Modern clinical 3T systems also allow rapid and accurate magnet shimming to correct for static field inhomogeneities. This meant that an entire MRI and ^1^H MRS placental examination could be obtained with a 40-minutes total acquisition time.

Although our study is limited by small numbers, we were able to detect a reproducible spectral output from placentae from all the women that were scanned, regardless of placental site and size, fetal motion and maternal habitus. However, for this proof-of-concept study we specifically recruited women with severe FGR who had evidence of fetal compromise and growth measurements <5^th^ centile. All babies with severe FGR had poor outcomes. To assess the clinical utility of ^1^H MRS as a diagnostic tool for placental failure, future studies should recruit women with less severe FGR to assess whether there is a lipid/choline ratio below which risk of adverse perinatal outcome increases.

In conclusion, our proof-of-concept study demonstrates that the MRS spectra of placentae in pregnancies complicated by severe FGR are significantly different from those from healthy pregnancies. Future studies should explore whether the absence of a choline peak represents a biomarker of critical placental failure and the consequence of this for perinatal outcome.
